# Integrative Analysis of Genomics and Transcriptome Data to Identify Regulation Networks in Female Osteoporosis

**DOI:** 10.3389/fgene.2020.600097

**Published:** 2020-11-30

**Authors:** Xianzuo Zhang, Kun Chen, Xiaoxuan Chen, Nikolaos Kourkoumelis, Guoyuan Li, Bing Wang, Chen Zhu

**Affiliations:** ^1^Department of Orthopedics, The First Affiliated Hospital of USTC, Division of Life Sciences and Medicine, University of Science and Technology of China, Hefei, China; ^2^College of Chemistry and Chemical Engineering, Xiamen University, Xiamen, China; ^3^Department of Medical Physics, School of Health Sciences, University of Ioannina, Ioannina, Greece; ^4^School of Electrical and Information Engineering, Anhui University of Technology, Ma'anshan, China

**Keywords:** osteoporosis, WGCNA (Weighted Gene Co-expression Network Analyses), pathway, biomarker, systems biology, LncRNA-long noncoding RNA

## Abstract

**Background:** Osteoporosis is a highly heritable skeletal muscle disease. However, the genetic mechanisms mediating the pathogenesis of osteoporosis remain unclear. Accordingly, in this study, we aimed to clarify the transcriptional regulation and heritability underlying the onset of osteoporosis.

**Methods:** Transcriptome gene expression data were obtained from the Gene Expression Omnibus database. Microarray data from peripheral blood monocytes of 73 Caucasian women with high and low bone mineral density (BMD) were analyzed. Differentially expressed messenger RNAs (mRNAs) and long non-coding RNAs (lncRNAs) were identified. Differences in BMD were then attributed to several gene modules using weighted gene co-expression network analysis (WGCNA). LncRNA/mRNA regulatory networks were constructed based on the WGCNA and subjected to functional enrichment analysis.

**Results:** In total, 3,355 mRNAs and 999 lncRNAs were identified as differentially expressed genes between patients with high and low BMD. The WGCNA yielded three gene modules, including 26 lncRNAs and 55 mRNAs as hub genes in the blue module, 36 lncRNAs and 31 mRNAs as hub genes in the turquoise module, and 56 mRNAs and 30 lncRNAs as hub genes in the brown module. *JUN* and *ACSL5* were subsequently identified in the modular gene network. After functional pathway enrichment, 40 lncRNAs and 16 mRNAs were found to be related to differences in BMD. All three modules were enriched in metabolic pathways. Finally, mRNA/lncRNA/pathway networks were constructed using the identified regulatory networks of lncRNAs/mRNAs and pathway enrichment relationships.

**Conclusion:** The mRNAs and lncRNAs identified in this WGCNA could be novel clinical targets in the diagnosis and management of osteoporosis. Our findings may help elucidate the complex interactions between transcripts and non-coding RNAs and provide novel perspectives on the regulatory mechanisms of osteoporosis.

## Introduction

Osteoporosis is a systemic disease of the musculoskeletal system. Its main pathophysiological characteristics are decreased bone mass, destruction of bone tissue microstructure, increased bone fragility, and increased fracture risk (Ensrud and Crandall, 2017). According to the National Health and Nutrition Examination Survey III, there are more than 9.9 million patients with osteoporosis in the United States of America, and 1.5 million patients suffer from osteoporotic fractures each year (Sahni et al., 2009). The social costs associated with osteoporosis are expected to rise as the population ages (Ruza et al., 2013). Affected by many factors, such as menopause, women are especially susceptible to osteoporosis (Baccaro et al., 2015). A large-scale epidemiological survey in 2006 showed that among people over 50 years old, the prevalence of osteoporosis in men was 14.4%, whereas that in women was as high as 20.7%(Chen et al., 2016). The lifetime risk of osteoporotic fractures in women is as high as 40%, which is significantly higher than the combined risks of breast cancer, endometrial cancer, and ovarian cancer (Ganji et al., 2019).

Osteoporosis is a disabling disease with insidious onset. In most patients, no symptoms are detected during the early to middle stages of illness. However, sudden osteoporotic fracture can lead to lifelong disability. Early detection and treatment can significantly improve survival rates and quality of life in patients with osteoporosis. However, our understanding of the pathogenesis of osteoporosis is not sufficient. Although many factors, such as oxidative stress (Zhou et al., 2016; Geng et al., 2019) and altered estrogen signaling (Sapir-Koren and Livshits, 2017), have been shown to contribute to osteoporosis, specific biomarkers for the early diagnosis and treatment of this disease have not yet been identified.

Despite the success of proteomics analyses for screening of molecular targets in osteoporosis (Xu et al., 2018; Saad, 2020), transcriptomic studies are now attracting much attention. Previous studies have shown that long non-coding RNAs (lncRNAs) are involved in the regulation of a series of biological processes, such as the occurrence and development of osteoporosis (Zhao et al., 2017; Zhou et al., 2019; Zhang et al., 2020). lncRNAs can directly interfere with messenger RNA (mRNA) transcription or form an endogenous competitive network with microRNAs (miRNAs) to regulate transcription (Zhang et al., 2020). The regulation mechanisms of lncRNA have been less studied compared with the more mature studies on miRNAs (Hupkes et al., 2014; You et al., 2016; Shao, 2017; Wang et al., 2018; Cui et al., 2019). Therefore, further research on the lncRNA/mRNA regulatory network in osteoporosis is needed for better dissemination.

Like most chronic diseases, osteoporosis is determined by a combination of genetic and environmental factors (Ongphiphadhanakul, 2007). The heritability of bone density is thought to be 50–85% (Ralston, 2010). However, all single genetic pathogenic factors discovered to date can explain <6% of heritability, including loci discovered by genome-wide association studies (GWASs) (Liu et al., 2014). In addition, the two-dimensional role of genes is limited. Therefore, building networks may improve our ability to discover the remaining heritability factors in patients with osteoporosis.

Most studies of osteoporosis have focused on screening for differentially expressed genes (DEGs) to identify biomarkers (Liu et al., 2014; Xia et al., 2017; Zhou et al., 2018a, 2019; Zhang et al., 2020). However, few studies have explored the relevance of genes that share a high degree of functional interconnection and are regulated in a similar fashion. Weighted gene co-expression network analysis (WGCNA), a systems biology method, is particularly useful in this context and may help establish free-scale gene co-expression networks to identify the associations between different gene sets or between gene sets and clinical features (Qian et al., 2019). Notably, WGCNA has been broadly used to identify hub genes linked with clinical features in different diseases, such as breast cancer (Li et al., 2019), heart failure (Niu et al., 2019), and osteoporosis (Farber, 2010; Chen et al., 2016; Zhang et al., 2016; Qian et al., 2019).

In the current study, WGCNA and other approaches were used to analyze microarray data from blood monocytes collected from pre-and postmenopausal women with low or high bone mineral density (BMD) to characterize the key genes associated with osteoporosis. We then constructed a regulatory network containing key mRNAs and lncRNAs based on the co-expression relationships. Our findings improve our understanding of the biological relationships between osteoporosis and genetics and identify novel potential gene targets for the diagnosis and treatment of osteoporosis.

## Methods

### Datasets and Samples

**Figure 1:**
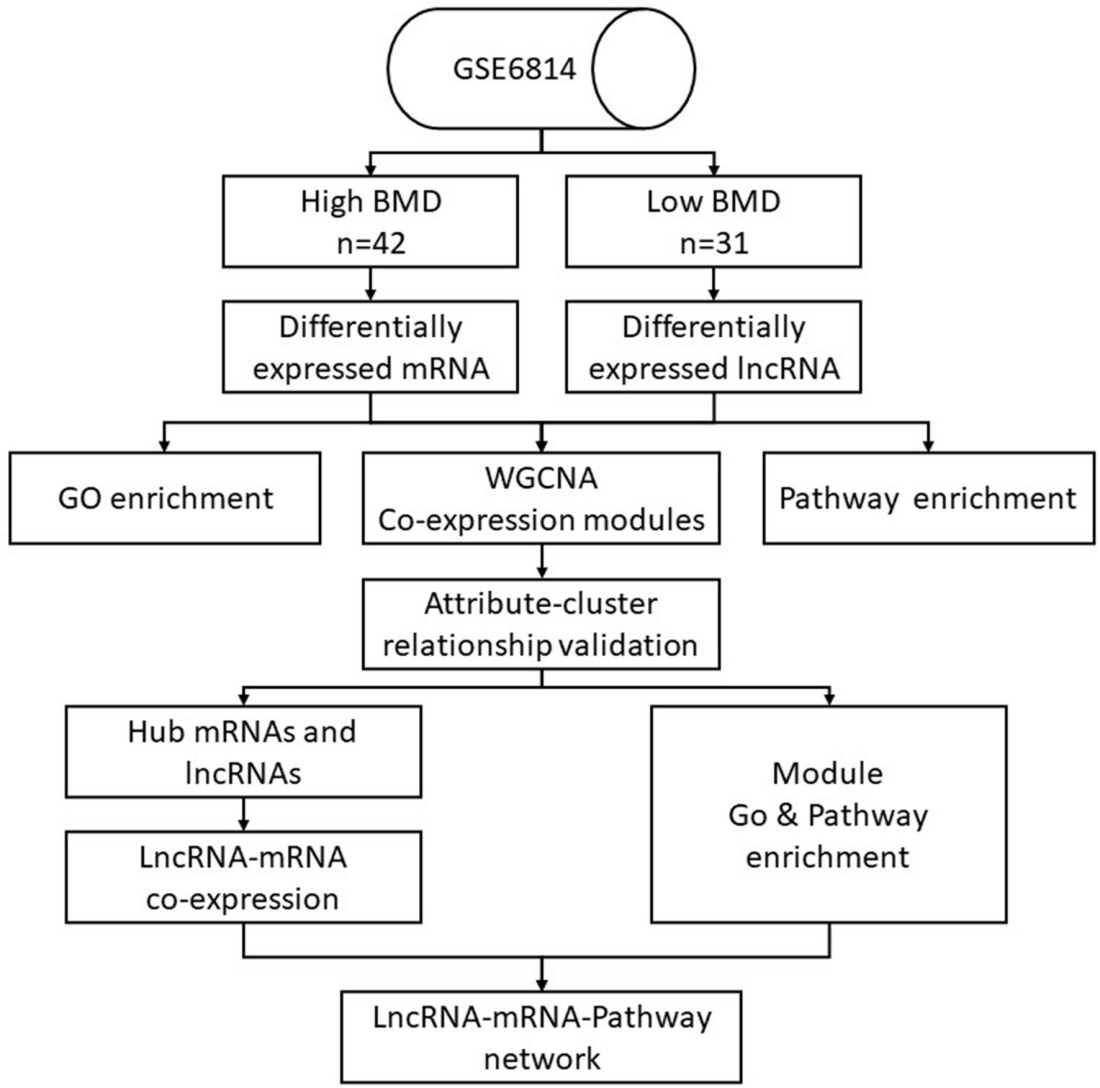
Data management flowchart of the study.

Data of this experiment are obtained and processed in the following ways (Figure 1). The microarray dataset GSE56814 was downloaded using the GEOquery package with R version (The R Foundation for Statistical Computing, Vienna, Austria) from the Gene Expression Omnibus (GEO) database (http://www.ncbi.nlm.nih.gov/geo/). The gene expression microarray was based on the GPL5175 platform (Affymetrix Human Exon 1.0 ST Array). Subjects for the study were enrolled in a previous microarray-based transcriptome-wide profiling study of peripheral blood monocytes in 73 Caucasian females (47–56 years old) (Liu et al., 2015). Briefly, the patients included 42 women with high BMD (aged 52.9 ± 2.3 years, Z-score = 1.38 ± 0.49) and 31 women with low BMD (aged 51.4 ± 2.6 years, Z-score = −1.05 ± 0.51; Table 1). The raw files of gene profiles were downloaded and processed with the Robust Multi-array Average (RMA) algorithm. The nsFilter algorithm was used to filter the data for the subsequent WGCNA.

**Table 1:** Demographic characteristics of the patient samples.

	**N**	**Age**	BMD^*^
High BMD	42	52.9 ± 2.3	1.38 ± 0.49
Low BMD	31	51.4 ± 2.6	−1.05 ± 0.51
Total	73	52.3 ± 2.4	0.34 ± 0.50

* *Hip BMD Z-score*.

### Annotation of lncRNAs From the Gene Expression Microarray Profile

LncRNAs were annotated from the gene expression microarray profile in two steps. First, we used the BLAST software to align the probes in GPL5175 to the mRNA database, which was selected from the overlap of coding RNAs in NCBI and Ensembl. Second, probes that could not be aligned to the mRNA database in the first step were further aligned to the lncRNA database, which included non-coding RNAs longer than 200 nucleotides collected from the NCBI, Ensembl, Refseq, and NONCODEv5 databases. Sequences were considered matching if they showed at least 90% identity. In both steps, the cutoff value was set to e-value <10e−5.

### Identification and Visualization of Differentially Expressed mRNAs and lncRNAs

A random variance model *t*-test, which could effectively increase the degrees of freedom for small samples, was used to filter differentially expressed mRNAs and lncRNAs between patients with high and low BMD (Wright and Simon, 2003). After significance and false discovery rate (FDR) analyses, we selected DEGs according to the *p* value threshold and absolute value of fold change (FC). Results with a *p* value of <0.05 with |FC| >1.2 were considered significantly different (Yang et al., 2005). For visualization, the differentially expressed mRNAs and lncRNAs were clustered using a hierarchical cluster algorithm with average linkage and Spearman's rank correlation distance, as provided by the EPCLUST software (http://ep.ebi.ac.uk/EP/EPCLUST/). Clustering was performed using the methods outlined in a previous publication (Misha et al., 2004). The results were visualized using heatmaps and dendrograms.

### Functional Enrichment Analysis

Gene ontology (GO) analysis, which organizes genes into hierarchical categories and uncovers gene regulatory networks based on biological processes and molecular functions, was used to analyze the main functions of DEGs (Gene Ontology, 2006). Kyoto Encyclopedia of Genes and Genomes (KEGG) pathway analysis was then used to identify the significant pathways for these genes (Kanehisa et al., 2004). The Database for Annotation, Visualization and Integrated Discovery (DAVID; https://david.ncifcrf.gov/) provides a comprehensive set of functional annotation tools to analyze high-throughput gene functions. GO and KEGG pathway enrichment analyses were performed using DAVID. We were only interested in biological processes and KEGG pathways showing significance according to the following parameters: *p* < 0.05, FDR <0.05, and enrichment score >1.5.

### WGCNA

WGCNA is an analysis method for complex samples and is used to mine module information from chip data (Wan et al., 2018). In the current study, WGCNA was performed using a freely accessible R package. To minimize the loss of statistical information, the top 25% of mRNAs from the absolute median deviation and the top 10% lncRNAs were selected for WGCNA. The Pearson coefficient between any two genes was calculated. Subsequently, the correlation coefficients took multiple powers of N so that the connections between genes in the network align with the scale-free network distribution. A one-step function was performed to construct the network and detect consensus modules. Additionally, we constructed a hierarchical clustering tree using the correlation coefficient between genes. Gene modules are indicated as different branches on the clustering tree, and different colors were used to distinguish the modules.

### Interaction Analysis of the Co-expression Modules

Interaction analysis of co-expression modules was performed as previously described Qian et al. (2019). Briefly, we calculated the eigengene adjacency based on similar co-expression in modules, and specific interactions among modules were evaluated using the flashClust function (Langfelder et al., 2012). A heatmap was established to elucidate the correlations among different modules.

### Construction of the lncRNA–mRNA Weighted Network

Using the modules obtained with WGCNA, hub genes were extracted as the top 100 genes in the module. Hub genes with high connectivity are usually regulatory factors located upstream of regulatory networks, whereas genes with low connectivity are usually located downstream of regulatory networks (e.g., transporters and catalytic enzymes). Thus, the co-expression relationships among hub genes were calculated, and the co-expression of lncRNAs/mRNAs among the top 50 hub genes, as well as the co-expression of mRNAs/mRNAs among the top 150 hub genes, was selected to construct a co-expression network. Interactions between lncRNAs and mRNAs were identified by calculating the Pearson correlation coefficient of differentially expressed mRNAs and lncRNAs with a cutoff |cor| >0.5. All interactions were identified using a p.adjust value <0.01. Next, lncRNA/mRNA regulatory networks were constructed using the Cytoscape software.

### Construction of the lncRNA/mRNA Pathway Weighted Co-expression Network

The lncRNA/mRNA pathway network was constructed based on the regulatory relationship of lncRNAs/mRNAs and the significant pathways involved in the regulation of mRNAs. The primary objective of this analysis was to identify the signaling pathways regulated by lncRNAs to predict possible mechanisms of lncRNAs in disease.

### Statistical Analysis

Data were analyzed using the SPSS 23.0 software (SPSS, Chicago, IL, USA). The random variance model *t*-test was performed using BRB-ArrayTools (v4.6, http://linus.nci.nih.gov/BRB-ArrayTools.html) (Wright and Simon, 2003). Because the sample size was limited, the adjusted *p* values were too large after multiple testing controls. We used a raw *p* <0.05 as the threshold for nominally significant differential expression. Notably, multiple testing adjustment with an FDR <0.05 was used to filter enriched GO and KEGG pathways.

## Results

### Differentially Expressed mRNAs and lncRNAs

**Figure 2:**
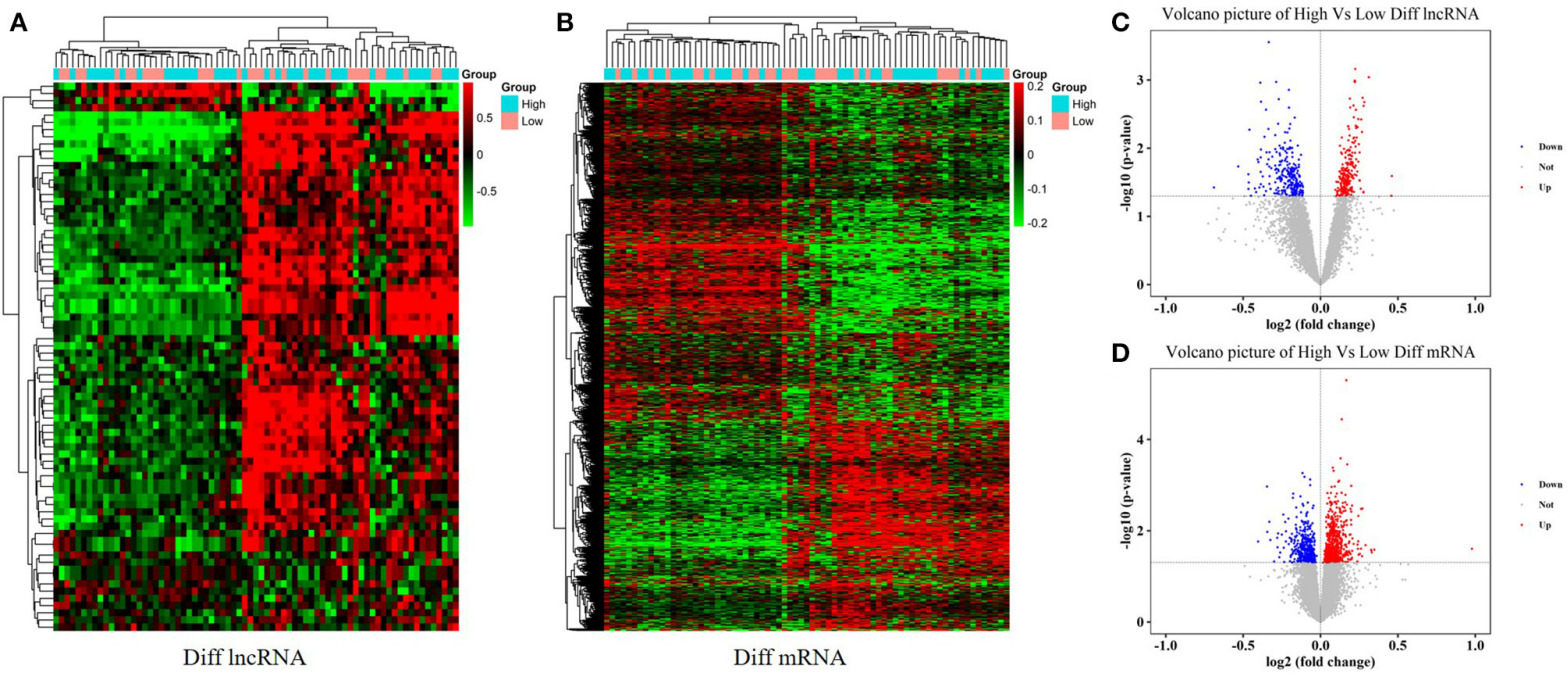
Differentially expressed mRNAs and lncRNAs between high and low hip BMD subjects. **(A,B)** The heatmaps represent hierarchical clustering for differentially expressed lncRNAs and mRNAs. **(C,D)** Volcano plots of significantly differently expressed genes (DEGs).

**Figure 3:**
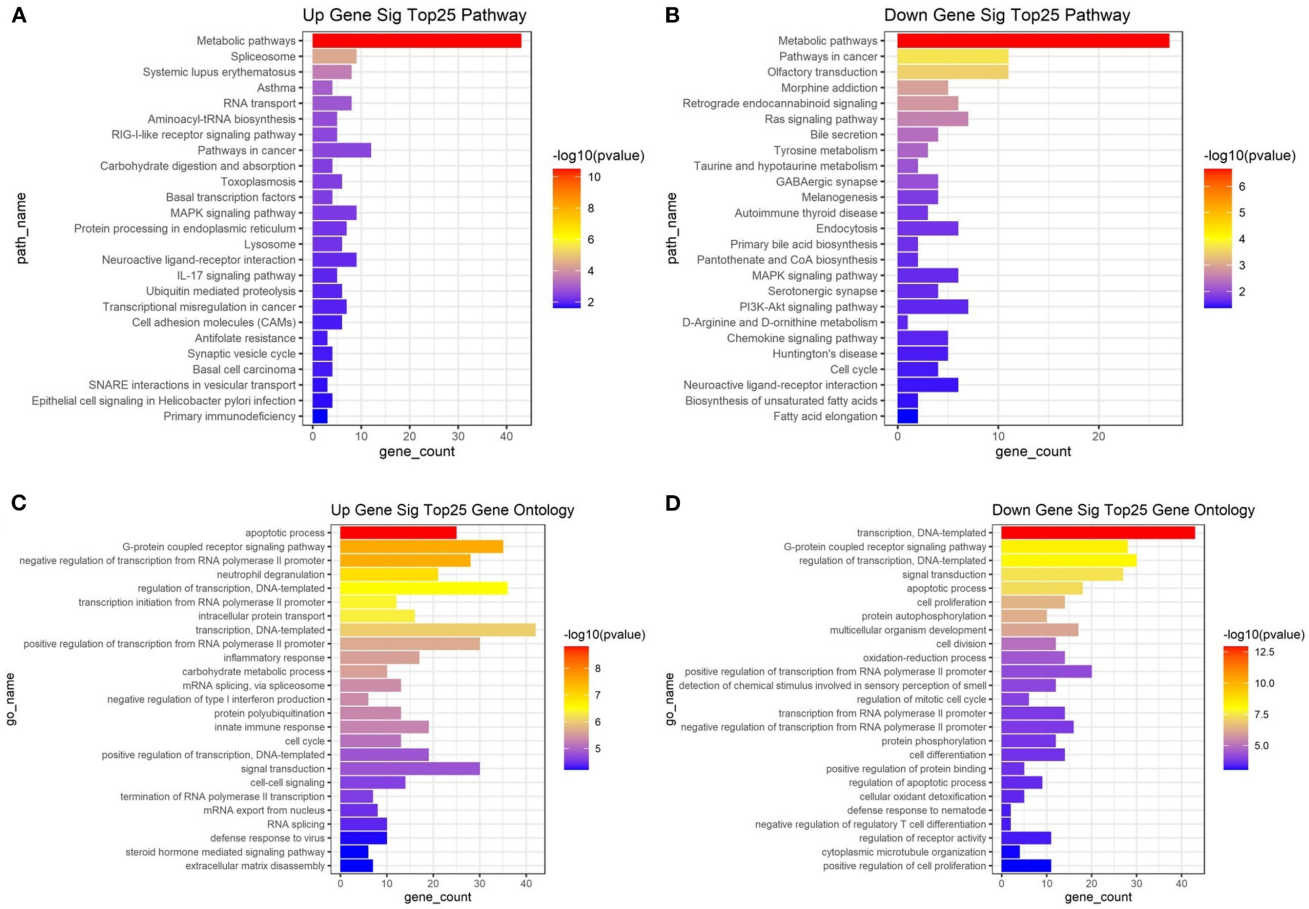
Kyoto Encyclopedia of Genes and Genomes (KEGG) pathway enrichment analysis of the top 25 up **(A)**/down **(B)**-regulated pathways enriched in differentially expressed genes between high/low BMD subjects. Top 25 up **(C)**/down **(D)**-regulated biological processes enriched in differentially expressed genes between high/low BMD subjects.

With an FC cutoff value >1.2 and *p* < 0.05, 3,355 mRNAs (Figures 2A,C) and 999 lncRNAs (Figures 2B,D) were identified as differentially expressed between patients with high and low hip BMD; these were selected as candidate genes for subsequent WGCNA. The pathway analysis reveals that the up-/down-regulated DEGs were primarily enriched in metabolic pathways (Figures 3A,B). The GO analysis found that up-regulated DEGs were enriched in terms of apoptotic process, G-protein coupled receptor signaling pathway, negative regulation of transcription from RNA polymerase II promoter, etc. Furthermore, the down-regulated ones were enriched in transcription, DNA-templated, G-protein coupled receptor signaling pathway, DNA-templated regulation of transcription, etc. (Figures 3C,D).

### Establishing Weighted lncRNA/mRNA Co-expression Networks and Identification of Soft Threshold Power

**Figure 4:**
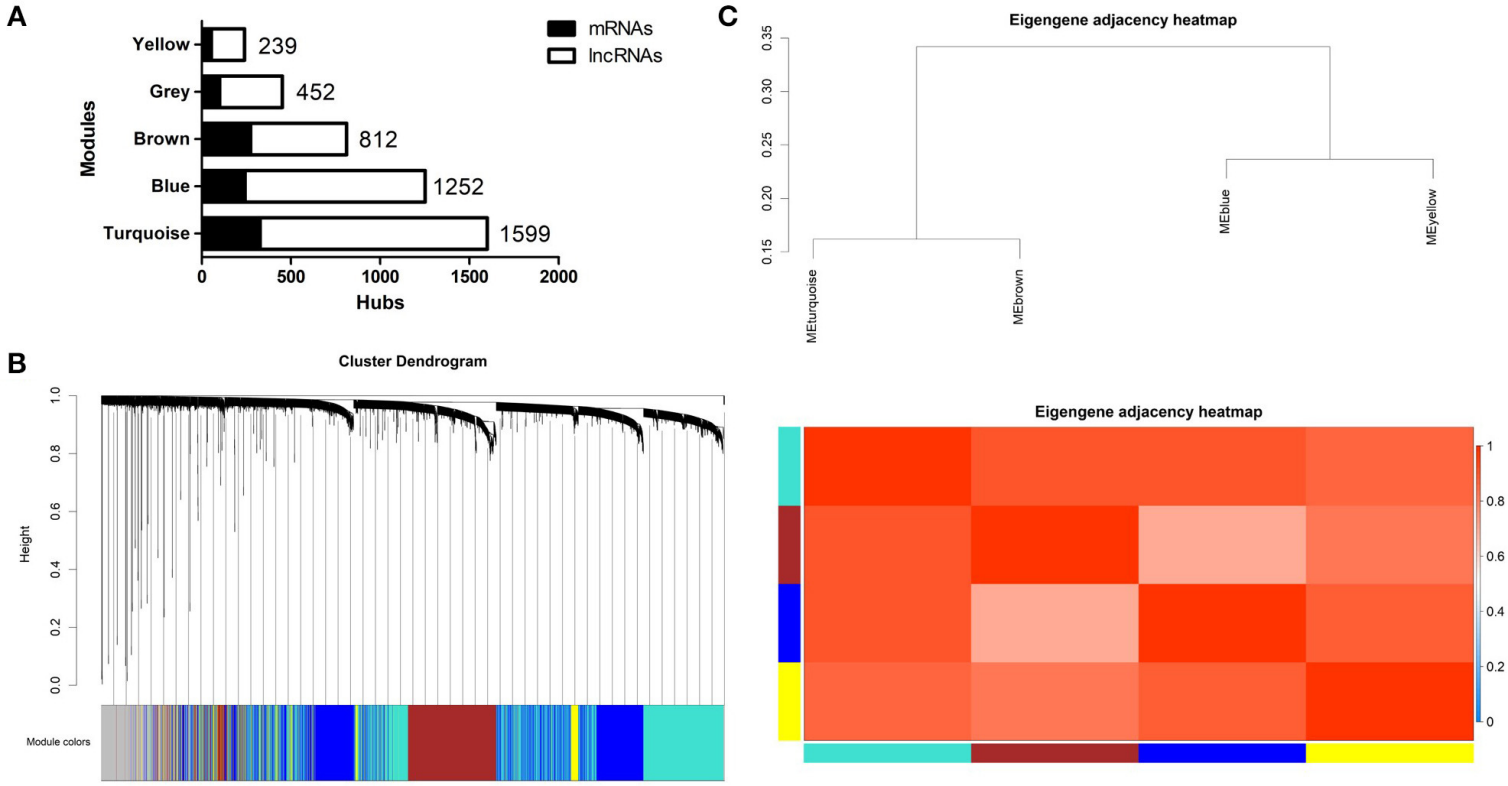
**(A)** Size and composition of the modules in the WGCNA network. **(B)** Dendrogram obtained by clustering the dissimilarity based on consensus topological overlap with the corresponding module colors indicated by the color row. **(C)** Eigengene adjacencies of heatmap. Red shows high adjacency.

A lncRNA/mRNA co-expression network was established from the newly generated set of mRNAs and lncRNAs. First, we performed cluster analysis on the selected mRNAs and lncRNAs. The results showed that no outlier existed in the sample; thus, there was no need to remove any outliers. Second, we used the R package to check the integrity of the data and constructed a network topology to determine the soft thresholding power. A soft threshold power of 6.5 was used to define the adjacency matrix, which was processed using the criteria of approximate scale-free topology. Third, the adjacent and topological matrices were obtained through the soft thresholding power. According to the topological matrix, genes were clustered through dissimilarity. Next, a dynamic shearing method was used to separate the cluster dendrogram into four modules, each indicated by a different color (turquoise, blue, brown, or yellow; gray was used for genes that did not fit into a distinct group). The largest module was the turquoise module, followed by the blue module. The size and composition of the modules are shown in Figure 4A. Of all selected genes, 351 mRNAs and 101 lncRNAs failed to fit within a distinct group and were assigned to the gray module (Figure 4B). After generating an eigengene adjacency heatmap (Figure 4C) to explore the correlations between modules, we found that the regulation directions of these modules were consistent. The modules showed a significantly positive correlation in patients with high BMD and a negative correlation in premenopausal women with low BMD. However, the correlation was not significant in postmenopausal women except that the gray module in patients with high BMD showed a correlation coefficient of 0.25 (*p* = 0.03).

### Functional Analyses and Pathway Enrichment of Different Modules

**Figure 5:**
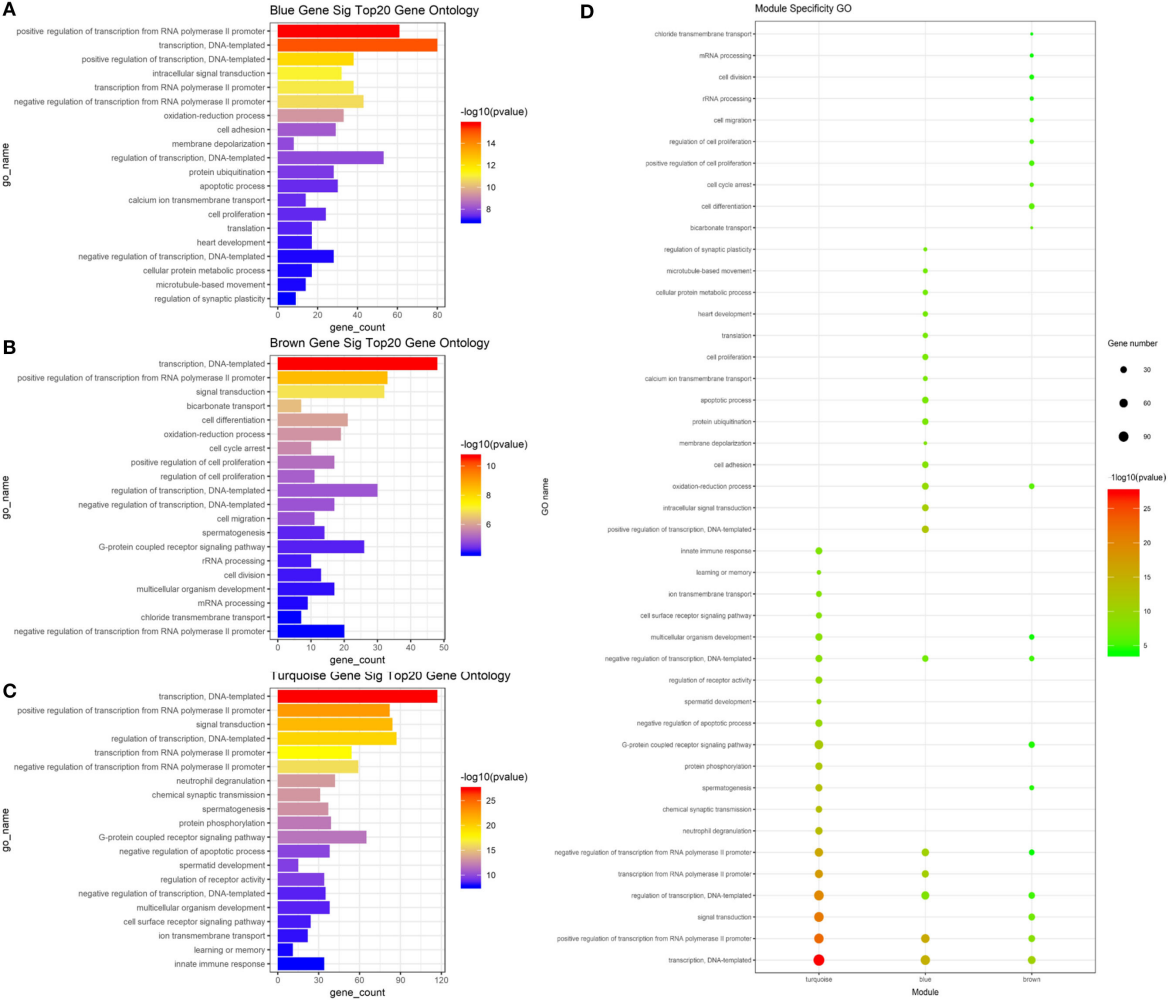
Result of GO analysis about mRNA based on WGCNA. **(A)** Top 20 enriched gene ontologies in blue module **(B)**. Top 20 enriched gene ontologies in brown module. **(C)** Top 20 enriched gene ontologies in turquoise module. **(D)** Module specificity gene ontologies. The vertical axis shows the results of GO analysis, and the horizontal axis shows the different modules. The color of the round shape shows transitional values (log10 of q value), and the size shows the number of genes that were enriched. mRNA, messenger RNA; WGCNA, weighted gene co-expression network analysis.

**Figure 6:**
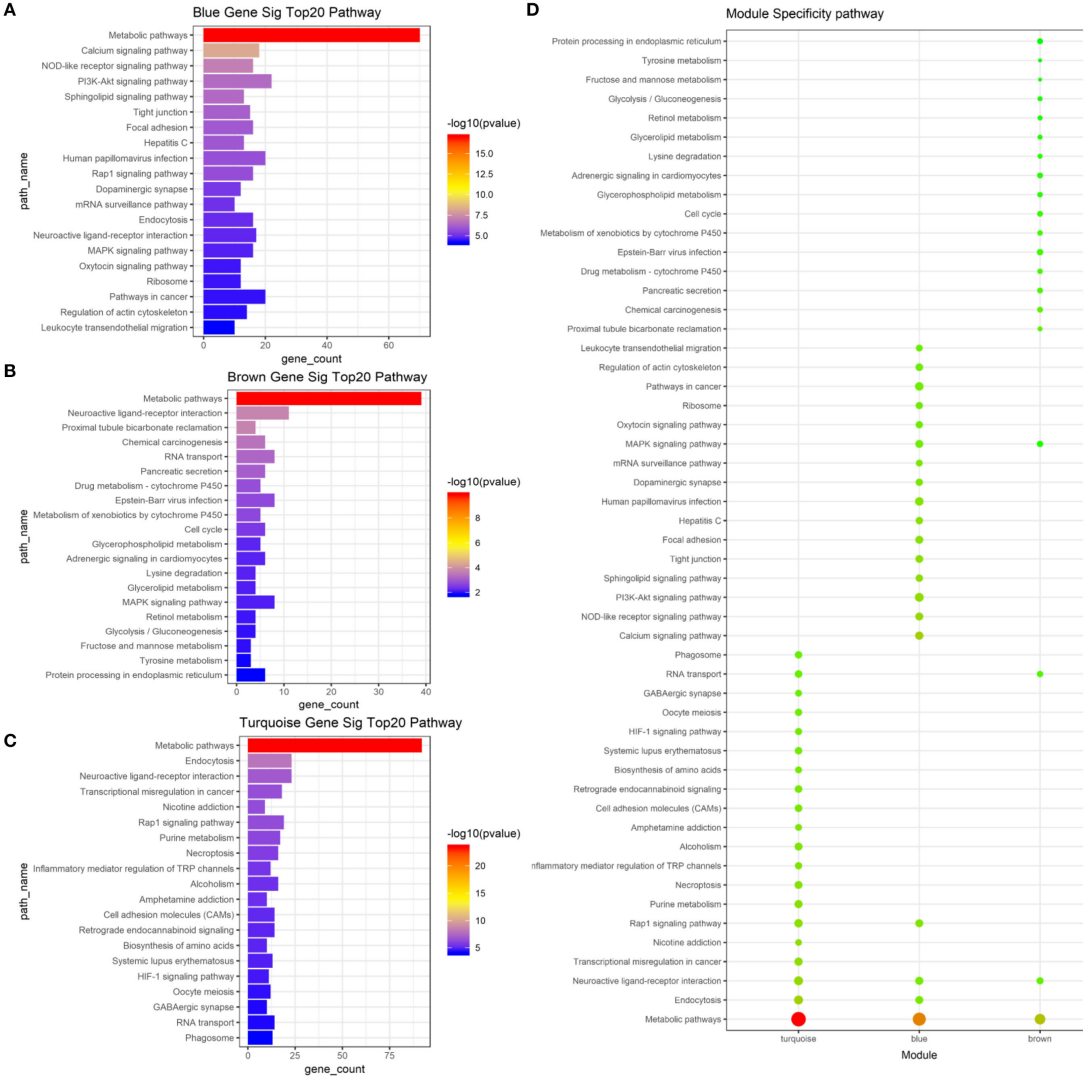
Result of pathway analysis about mRNA based on WGCNA. **(A)** Top 20 enriched pathways in blue module **(B)**. Top 20 enriched pathways in brown module. **(C)** Top 20 enriched pathways in turquoise module. **(D)** Module specificity pathways. The vertical axis shows the results of pathway analysis, and the horizontal axis shows the different modules. The color of the round shape shows transitional values (log10 of q value), and the size shows the number of genes that were enriched. mRNA, messenger RNA; WGCNA, weighted gene co-expression network analysis.

To determine whether the modules were composed of functionally similar genes and to understand the functional significance of the network modules, GO term and KEGG pathway enrichment analyses were performed. The enrichment results from the yellow module were not significant because there were few genes in this module. The GO results of all three modules were enriched in the positive regulation of transcription from RNA polymerase II promoter, DNA-templated transcription, and their regulatory mechanisms. Specifically, genes in the blue module were highly enriched in cell surface receptor signaling pathway, chemical synaptic transmission, ion transmembrane transport, multicellular organism development, neutrophil degranulation, and regulation of receptor activity. The turquoise module was associated with calcium ion transmembrane transport, cell adhesion, cell proliferation, cellular protein metabolic process, membrane depolarization, and microtubule-based movement. The brown module was associated with bicarbonate transport, cell cycle arrest, cell differentiation, cell division, cell migration, oxidation–reduction process, and rRNA processing. The top 20 GO terms for the three modules are shown in Figure 5. mRNA pathway enrichment was also analyzed. Notably, all three modules were significantly enriched in metabolic pathways and neuroactive ligand–receptor interactions. The turquoise module was specifically associated with purine metabolism, necroptosis, inflammatory mediator regulation of TRP channels, alcoholism, and the hypoxia-inducible factor-1 signaling pathway. The blue module was associated with the Rap1 signaling pathway, calcium signaling pathway, NOD-like receptor signaling pathway, phosphatidylinositol 3-kinase/Akt signaling pathway, and sphingolipid signaling pathway. The brown module was associated with protein processing in the endoplasmic reticulum, tyrosine metabolism, glycerophospholipid metabolism, cell cycle, and metabolism of xenobiotics by cytochrome P450. The top 20 pathways for each module are shown in Figure 6.

### WGCNA Hub Gene Identification

**Figure 7:**
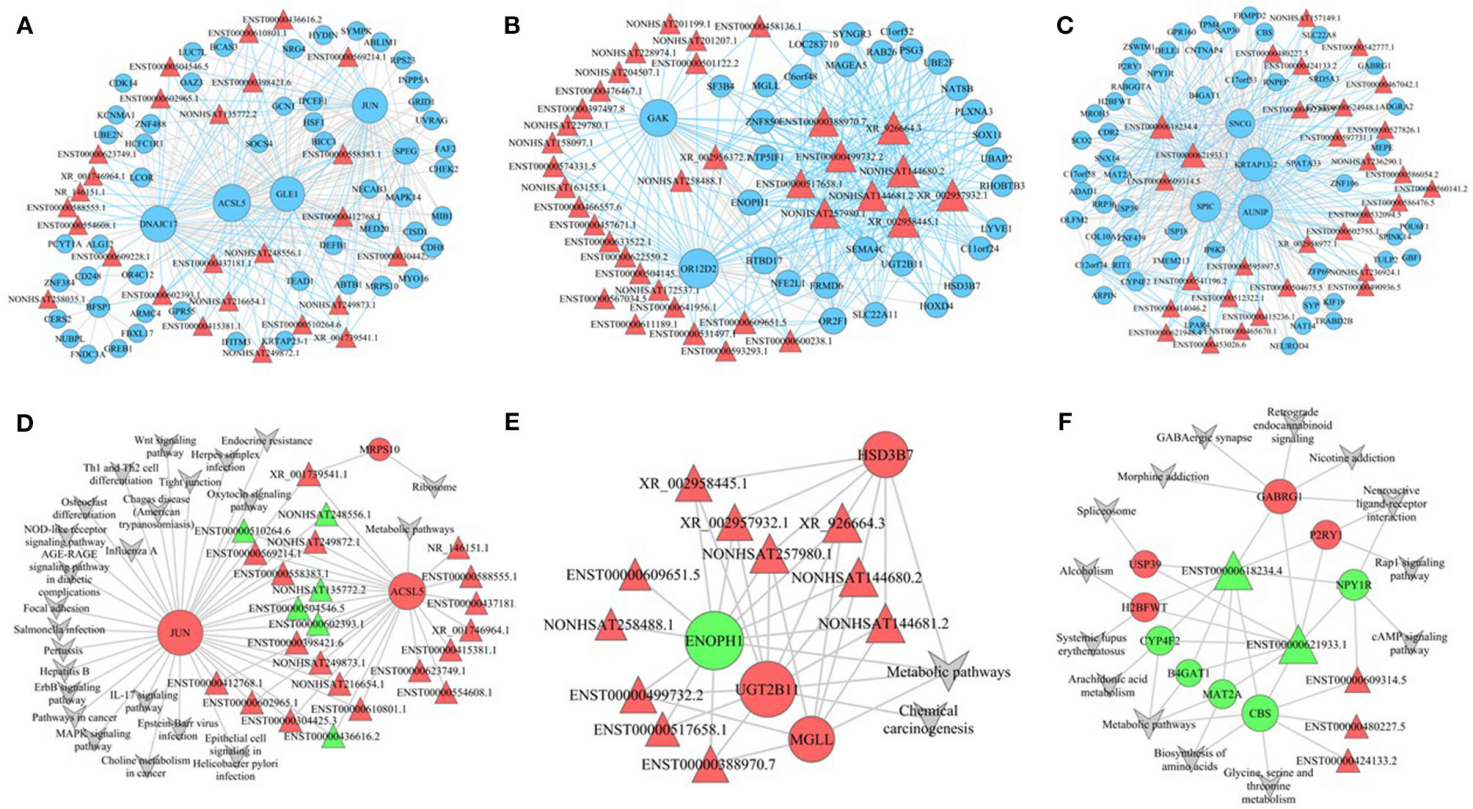
Established lncRNA–mRNA network and lncRNA–mRNA pathway. **(A–C)** lncRNA–mRNA network of genes in blue **(A)**, brown **(B)**, and turquoise **(C)** modules. **(D–F)** lncRNA–mRNA pathway of genes in blue **(D)**, brown **(E)**, and turquoise **(F)** modules.

Hub genes are usually key regulators, such as transcription factors, and are worthy of in-depth analysis and mining. In the blue module, we found 26 lncRNAs and 55 mRNAs as hub genes (Figure 7A). We analyzed the functions of these hub genes and found that these genes were mainly involved in response to muscle stretch (e.g., *JUN* and *MAPK14*), biotic stimulus (e.g., *IFITM3*), and ventricular system development (e.g., *HYDIN* and *ARMC4*). The cell components were enriched in the cytoplasm (e.g., *BCAS3, CD248, DNAJC17, GCN1*, and *GLE1*), endoplasmic reticulum (e.g., *ALG12, NECAB3, UVRAG, CERS2*, and *KCNMA1*), and endoplasmic reticulum membrane (e.g., *ALG12, NECAB3, CERS2*, and *PCYT1A*). The molecular functions were mainly enriched in ubiquitin protein ligase binding (e.g., *FAF2, ABTB1*, and *UBE2N*). We also observed 36 lncRNAs and 31 mRNAs as hub nodes in the turquoise module (Figure 7B) and 56 mRNAs and 30 lncRNAs as hub nodes in the brown module (Figure 7C).

### Construction of lncRNA/mRNA Pathway Co-expression Networks

To uncover the possible mechanisms of lncRNA-mediated regulation of signaling pathways, we selected a number of pathways with significant differences in the turquoise, blue, and brown modules and associated them with the lncRNA/mRNA co-expression network. In the pathway co-expression network, the blue module had 3 mRNAs and 24 lncRNAs (Figure 7D), the brown module had 4 mRNAs and 11 lncRNAs (Figure 7E), and the turquoise module had 9 mRNAs and 5 lncRNAs (Figure 7F). In the blue module, XR_001739541.1 was linked to *MRPS10, ACSL5*, and *JUN* and was therefore enriched in the ribosome pathway and metabolic pathway. Sixteen lncRNAs, including NONHSAT249872.1 and ENST00000510264.6, were linked to two mRNAs (*JUN* and *ACSL5*) and were enriched in pathways, such as the NOD-like receptor signaling pathway, mitogen-activated protein kinase signaling pathway, Wnt signaling pathway, ErbB signaling pathway, osteoclast differentiation, and metabolic pathways. Seven other lncRNAs were linked to *ACSL5* and were enriched in metabolic pathways. In the brown module, XR_002958445.1, XR_002957932.1, NONHSAT257980.1, XR_926664.3, NONHSAT144580.2, and NONHSAT144681.2 were connected to *HSD3B7, ENH1, UGT2B11*, and *MGLL* mRNAs and were therefore enriched in metabolic pathways and chemical carcinogenesis. In the turquoise module, ENST00000618234.4 and ENST00000621933.1 were linked to nine mRNAs (*USP39, H2BFWT, CYP4F2, B4GAT1, MAT2A, CBS, GABRG1, P2RY1*, and *NPY1R*) and enriched in pathways, such as GABAergic synapse, retrograde endocannabinoid signaling, Rap1 signaling pathways, and cAMP signaling pathway. The lncRNAs ENST00000609314.5, ENST00000480227.5, and ENST00000424133.2 were also involved in the turquoise modular lncRNA/mRNA pathway co-expression network.

## Discussion

Osteoporosis is a common and complex systemic bone disease, and women are especially susceptible to this disease. The onset of osteoporosis is insidious, and the disease often remains undetected in the early stages. However, once a secondary osteoporotic fracture occurs, many complications can occur, and the prognosis is poor. Therefore, many researchers have investigated the molecular diagnosis, treatment targets, and genetic regulation of osteoporosis. In a previous study, Liu showed that *DAXX* and *PLK3*, which are related to induction of apoptosis, were down-regulated in patients with a low BMD among a cohort of 73 Caucasian females (Liu et al., 2015). Based on the same microarray dataset available online, Zhou performed GWAS and found 29 potential transcription factors for up-regulated genes and 9 transcription factors for down-regulated genes (Zhou et al., 2018a). They further investigated the relationships between mRNAs and lncRNAs using two approaches and claimed that 26 candidate lncRNAs may regulate mRNA expression (Zhou et al., 2019). After correcting for crosstalk effects, they identified several significant enriched pathways involved in BMD regulation (Zhou et al., 2018b). Moreover, Xia established a meta-analysis using the microarray datasets GSE56815 and GSE56814 and found 10 potential pathogenic genes of osteoporosis (Xia et al., 2017).

In this study, we found 4,354 DEGs in the peripheral blood chips of patients with high or low BMD in the hip; these included 3,355 mRNAs and 999 differentially expressed lncRNAs. In contrast to previous studies based on protein–protein interaction (PPI) networks, we employed WGCNA to aggregate genes with common expression characteristics into modules. This systemic biology method helped free-scale gene co-expression networks to identify associations without previous PPI knowledge (Zheng et al., 2020). The WGCNA co-expression networks revealed three gene modules consisting of 40 lncRNAs and 16 mRNAs, which were significantly related to the level of BMD. In a previous study, Qian (Qian et al., 2019) found 12 genes as hub genes in 80 Caucasian females. Another WGCNA study identified six genes from 26 healthy young Chinese females (Farber, 2010). Zhang et al. found seven genes that were significantly down- or up-regulated using traditional comparative analysis, WGCNA, and gene set enrichment analysis (Zhang et al., 2016). Chen constructed a WGCNA co-expression network composed of BMD GWAS genes and found two functional gene modules and nine interesting genes. Of note, the genes identified in the current study did not overlap in these previous studies. We attribute the observed discrepancy to differences in patients and ethnicity; these potential differences should be investigated further. The differentially expressed mRNAs and lncRNAs were primarily involved in metabolic pathways, including glycerophospholipid metabolism, lysine degradation, and glycerolipid metabolism.

Our study and previous studies have established possible targets for the treatment of osteoporosis, such as JUN (Ralston, 2010; Zhou et al., 2019). JUN belongs to the AP-1 family of transcription factors, which includes c-Fos, Fra1, Fra2, JunB, and JunD. JUN expression was significantly up-regulated in dental pulp stem cells induced to undergo osteogenic differentiation (Guo et al., 2018). Higher concentrations of glucocorticoids impair osteogenesis by inhibiting JUN expression and human bone marrow mesenchymal stem cell (BMSC) proliferation, which can be driven by glucocorticoid receptor and AP-1 crosstalk (Carcamo-Orive et al., 2010). Moreover, our recent study showed that JUN can drive bone formation by expanding osteoprogenitor populations and forcing them into the bone fate, providing a rationale for future clinical applications (Lerbs et al., 2020).

Long-chain fatty acyl-CoA synthetases 5 (ACSL5) is an isozyme of the long-chain fatty-acid-coenzyme A ligase family. It is a regulatory enzyme that converts free long-chain fatty acids into fatty acyl-CoA esters and thereby plays key roles in lipid biosynthesis and fatty acid degradation. Currently, there is no evidence that ACSL5 expression is involved in osteoporosis; however, the presence of ACSL5 is obviously related to disorders of glucose metabolism. High glucocorticoid concentrations impair osteogenesis (Carcamo-Orive et al., 2010) and induce the activation of osteoclast proliferation and differentiation (Wongdee and Charoenphandhu, 2011). In addition, ACSL5 may also be an important mediator in apoptosis (Xia et al., 2016). Further studies are needed to assess the potential roles of this protein in the pathophysiological process of osteoporosis.

The lncRNA/mRNA regulatory networks were further constructed using high connectivity hub genes in the WGCNA co-expression network. Compared with nodes with low connectivity, nodes with high connectivity play more important roles in the entire transcription network and are more likely to be upstream regulators. According to the above-mentioned regulatory relationships of lncRNAs/mRNAs and the significantly involved pathways, we further constructed a network of pathways in which lncRNAs could regulate mRNAs through co-expression and thereby play roles in these pathways. Notably, metabolic pathways were significantly enriched in all three functional gene modules. Bone formation is known to be dependent on the supply of metabolites to monocytes in the bone marrow (Bidwell et al., 2013). Additionally, the balance of bone metabolism depends on the coordination of bone formation and resorption, and this process requires information exchange between different types of cells. For example, the lncRNA *Bmncr* is a key regulator of age-related osteogenic niche alteration and cell fate switch of BMSCs (Li et al., 2018). Moreover, the lncRNA *ODSM* functions as a competing endogenous RNA in the lncRNA *ODSM*/*miR-139-3p*/*ELK1* pathway and has important functions in osteoblast differentiation and apoptosis (Wang et al., 2018). Further studies are needed to explore the molecular mechanisms through which lncRNAs act as transcription factors to regulate osteoporosis (Zhang et al., 2020). It is worth noting that the above-mentioned molecular targets may be indirectly related to the BMD phenotype. In the process of establishing the aforementioned weighted lncRNA/mRNA co-expression networks, there was no observed direct quantitative relationship with the level of BMD. These genes aggregate to form modules through co-expression relationships. They have significant correlations and may participate in certain biological processes together. Not all genes in these modules are directly related to the level of BMD, which makes it difficult for us to interpret the experimental results within the context of BMD levels. Therefore, it is necessary to construct a network relationship, find the hub genes, and conduct further *in vitro* validations.

There were some limitations to this study. First, this study was based purely on microarray datasets, and we did not obtain any data directly from *in vivo* experiments. Thus, further studies are needed to confirm the observed molecular mechanisms. Second, when selecting the phenotype of osteoporosis, we used BMD as the only indicator. Because phenotype identification can directly influence patient grouping and is crucial to the construction of gene networks, additional indicators (e.g., bone geometric parameters, bone size, and compressive strength index of the femoral neck) should be evaluated in further studies in order to obtain a complete picture of osteoporosis. Third, this study did not compare the obtained results in female osteoporosis with male cases, because there are few samples of male osteoporosis in the public database, and the platforms are not the same. It is worth noting that the above-mentioned biomarkers were all found in female database samples; therefore, we may not be able to extrapolate these conclusions to samples of male patients. Previous studies have shown that miRNAs are gender-dependent as molecular targets of BMD (Kelch et al., 2017). Finally, this study is based on gene expression from blood monocytes. This is far removed from therapeutic application in musculoskeletal diseases. Further validation on bone samples should be done in future research.

In conclusion, in this study, we identified differentially expressed mRNAs and lncRNAs in existing microarray profile data. A WGCNA was constructed and yielded three significant modules associated with differences in BMD. Enrichment analysis indicated that the modules were primarily enriched in metabolic pathways, such as glycerophospholipid metabolism, lysine degradation, and glycerolipid metabolism. Several hub genes, including *JUN* and *ACSL5*, were found and may represent potential biomarkers or clinical targets for osteoporosis. In addition, a comprehensive lncRNA/mRNA-pathway regulatory network was built to elucidate the complex interactions between the transcripts and non-coding RNAs. Our findings provided a novel perspective on the regulatory mechanisms of osteoporosis.

## Data Availability Statement

Publicly available datasets were analyzed in this study. This data can be found here: <GEO data base (http://www.ncbi.nlm.nih.gov/geo/) GSE56814>.

## Ethics Statement

The studies involving human participants were reviewed and approved by the ethics committee of the First Affiliated Hospital of USTC. The patients/participants provided their written informed consent to participate in this study.

## Author Contributions

XZ conceived the idea and designed the project. XC performed the data analysis. XZ and KC wrote the paper. NK, GL, and BW revised the manuscript. GL and CZ gained institutional funding. CZ provided administrative support. All authors have read and approved the manuscript.

## Conflict of Interest

The authors declare that the research was conducted in the absence of any commercial or financial relationships that could be construed as a potential conflict of interest.
